# Adjusting the Cut-Off and Maximum Pool Size in RT-qPCR Pool Testing for SARS-CoV-2

**DOI:** 10.3390/v13040557

**Published:** 2021-03-26

**Authors:** Murilo S. Costa, Hugo I. Sato, Raissa P. Rocha, Alex F. Carvalho, Nathalia S. Guimarães, Elaine L. Machado, Claudia R. L. Alves, Santuza M. R. Teixeira, Ricardo H. C. Takahashi, Unaí Tupinambás, Flávio G. da Fonseca

**Affiliations:** 1 Graduate Program in Infectology and Tropical Medicine, Medical School, Universidade Federal de Minas Gerais, Belo Horizonte 30130-100, MG, Brazil; murilosc@ufmg.br; 2 Vaccine Technology Center, Universidade Federal de Minas Gerais, Belo Horizonte 31310-260, MG, Brazil; hugosato@ufmg.br (H.I.S.); raissa-biotec@ufmg.br (R.P.R.); alexficar@gmail.com (A.F.C.); santuzat@ufmg.br (S.M.R.T.); 3 Graduate Program in Health and Nutrition, Universidade Federal de Ouro Preto, Ouro Preto 35400-000, MG, Brazil; nathalia.guimaraes@aluno.ufop.edu.br; 4 Department of Preventive and Social Medicine, Medical School, Universidade Federal de Minas Gerais, Belo Horizonte 30130-100, MG, Brazil; elainelm@ufmg.br; 5 Department of Pediatrics, Medical School, Universidade Federal de Minas Gerais, Belo Horizonte 30130-100, MG, Brazil; lindgren@ufmg.br; 6 Department of Mathematics, Universidade Federal de Minas Gerais, Belo Horizonte 31270-901, MG, Brazil; taka@mat.ufmg.br; 7 Department of Internal Medicine, Medical School, Universidade Federal de Minas Gerais, Belo Horizonte 30130-100, MG, Brazil; unai@ufmg.br

**Keywords:** SARS-CoV-2, COVID-19, pool testing, RT-qPCR

## Abstract

Reverse transcription quantitative real-time polymerase chain reaction (RT-qPCR) to detect SARS-CoV-2 RNA is an essential test to monitor the occurrence of COVID-19. A methodology is proposed for the determination of maximum pool size and adjustments of cut-off values of cycle threshold ($C_{t}$ in RT-qPCR pool testing, to compensate for the dilution caused by pooling. The trade-off between pool size and test sensitivity is stated explicitly. The procedure was designed to ensure that samples that would be detectable in individual testing remain detectable in pool testing. The proposed relaxation in cut-off is dependent on the pool size, allowing a relatively tight correction to avoid loss of detection of positive samples. The methodology was evaluated in a study of pool testing of adults attending a public emergency care unit, reference for COVID-19 in Belo Horizonte, Brazil, and presenting flu-like symptoms. Even samples on the edge of detectability in individual testing were detected correctly. The proposed procedure enhances the consistency of RT-qPCR pool testing by enforcing that the scales of detectability in pool processing and in individual sample processing are compatible. This may enhance the contribution of pool testing to large-scale testing for COVID-19.

## 1. Introduction

Most SARS-CoV-2-infected patients either present symptoms indistinguishable from other flu-like syndromes or remain asymptomatic. Therefore, the effective control of the epidemics requires testing large numbers of people regularly. The reverse transcription quantitative real-time polymerase chain reaction (RT-qPCR) is the most sensitive test for detecting SARS-CoV-2 shortly after the infection. However, its widespread application is limited by the cost of reagents, the need for specific laboratory equipment, sample transport logistics, and the long processing time [1,2,3].

Pool testing is a method of grouping samples to be tested together, to reduce costs and quicken the process. The number of samples on each pool should vary according to the infection’s prevalence [1,2,3]. If viral RNA is detected in a pool, each sample must be tested individually to identify the virus-positive ones. If the virus is not detected, all samples are considered non-detectable [1,2,3]. Pool testing allows substantial savings in test average cost and average time for delivering results. These characteristics are especially relevant to expand the testing coverage in scenarios with limited resources. As a shortcoming, if a sample with low viral load is included in a pool with negative samples, the reduced concentration of viral RNA in the pool can produce a false-negative result [4,5].

The usual criterion for discrimination between positive and negative results is based on the definition of a maximum value for the $C_{t}$ (cycle threshold) necessary to amplify the viral RNA in the sample up to a detectable level. Samples that become detectable with a $C_{t}$ greater than a pre-determined cut-off value ${\bar{C}}_{t}$ are interpreted as negative since the detected fluorescence is likely to represent noise. The authors of Reference [5] report that, in their experiments, the $C_{t}$ value in pooled samples is increased by a factor of 1.24 for each increase of dilution by a factor of 2. Other works also report experiments in which changes of the $C_{t}$ value in pooled samples are observed [6,7]. Although the authors of Reference [6] advise the “need for careful experimental design” due to that effect, no explicit guidelines for adapting the detection procedures for pool testing are provided in any of those works.

The goal of this study was to propose a methodology to determine the maximum pool size and to adjust cut-off values of cycle threshold ($C_{t}$) in RT-qPCR pool testing in order to compensate for the dilution caused by pooling. This article is organized as follows: A procedure is presented for the estimation of a lower bound $\bar{\gamma }$ for the amplification factor γ, which is characteristic of each specific laboratory setting. Then, a formula for the cut-off value of cycle threshold to be applied in pooled samples, denoted by ${\bar{C}}_{t}^{*}$, is developed as a function of the pool size N, of $\bar{\gamma }$, and of the cut-off value adopted in individual testing, ${\bar{C}}_{t}$. This formula gives the cut-off that would be necessary for detecting viral RNA, in a pool testing, in a sample situated in the edge of detectability of individual testing. As the value of ${\bar{C}}_{t}^{*}$ may become greater than the upper limit of the equipment detection range, $C_{M}$, as the pool size grows, then an upper bound $\bar{N}$ for the pool size N is established, as a function of ${\bar{C}}_{t}$, $C_{M}$, and $\bar{\gamma }$, to guarantee that the sensibility of pool testing remains compatible with individual testing. The results of a calibration assay for the determination of $\bar{\gamma }$ in the specific laboratory setting used in this study are presented, and the respective specific formulae for ${\bar{C}}_{t}^{*}$ and $\bar{N}$ are derived. Finally, the application of the overall procedure in the processing of pooled samples collected from patients that attended a public emergency care unit are discussed.

## 2. Materials and Methods

### 2.1. Ethical Approval

This study was conducted within the preliminary phase of the research project Evaluation of the COVID-19 Diagnosis in Patients with Flu-Like Syndromes Attended at the Specialized COVID-19 Centers in Belo Horizonte, MG, Brazil. The research was approved by the UFMG Ethics Committee, CAAE-35074720.3.0000.5149, 23 June 2020.

### 2.2. Amplification Factor Estimation

In RT-qPCR, the concentration *P* of viral RNA in an individual sample after *C* cycles of replication is given by:

(1) $$P=P_{0}\gamma^{C}$$

with *P*_0_ representing the initial concentration before starting replication, and γ representing the amplification factor per cycle. Let $C_{t}$ represent the number of cycles that amplifies *P* up to the detection threshold. If the positive sample is diluted in a pool with (*N*-1) negative samples, there will be a reduction of the initial viral concentration by a factor *N*. Therefore, the increase in the number of cycles for reaching detection in a pool is given by:

(2) $$∆\;=\;C_{t}^{*}\;-\;C_{t}=\frac{\ln N}{\ln\gamma }$$

with $C_{t}^{*}$ representing the number of cycles needed to reach the detection threshold in pool testing. For a given γ, different pool sizes lead to different Δ increments. For a specific set of reagents and PCR instrument, an explicit formula for the amplification factor γ can be stated:

(3) $$\gamma \;=\;N^{\frac{1}{∆}}$$

The apparent value of parameter γ will vary between different runs, in the same equipment, due to random differences in relative volumes of reagent and sample material, and in relative volumes of different samples in a pool. The least reasonable value of γ should be employed in Equation (2), such that the relaxation Δ becomes greater than the expected empirical values of $C_{t}^{*}$ found in practice.

### 2.3. Cut-Off Adaptation for Pooled Samples

Let ${\bar{C}}_{t}$ denote the cut-off value adopted in individual sample testing (any $C_{t}$ value above it is assumed to indicate non-detection). The cut-off value ${\bar{C}}_{t}^{*}$ for pooled samples that allows the detection of samples that are on the edge of detection in individual testing, with $C_{t}\;=\;{\bar{C}}_{t}$, should be:

(4) $${\bar{C}}_{t}^{*}\;=\;{\bar{C}}_{t}+\frac{\ln\left(N\right)}{\ln\left(\bar{\gamma }\right)}$$

### 2.4. Pool Size Upper Bound

Each laboratory setting detection range which is bounded by a maximum detectable $C_{t}$ value, denoted as $C_{M}$, needs to be determined. Using cut-off values above this limit would lead to false-positive results. Therefore, if the testing is expected to detect viral RNA in samples with $C_{t}$ up to ${\bar{C}}_{t}$, the pool size *N* must be chosen such that ${\bar{C}}_{t}^{*}\;\leq \;C_{M}$, which establishes an upper bound $\bar{N}$ for the pool size that can be used:

(5) $$N\;\leq \;\bar{N}\;=\;exp\left[\left(C_{M}\;-\;{\bar{C}}_{t}\right)\ln\left(\bar{\gamma }\right)\right]\;$$

Assuming that $C_{M}$ and $\bar{\gamma }$ are fixed values, intrinsic to the specific laboratory setting, Equation (5) quantifies the trade-off between the maximum pool size $\bar{N}$ and the target detection threshold ${\bar{C}}_{t}$. A stringent requirement on the detection of samples with high $C_{t}$ is translated in high ${\bar{C}}_{t}$ values, leading to small pools. As that requirement is relaxed to smaller values of ${\bar{C}}_{t}$, larger pool sizes become admissible, at the cost of possibly not detecting samples with $C_{t}$ greater than ${\bar{C}}_{t}$.

### 2.5. RT-qPCR Testing Framework

All testing experiments reported here were conducted in the CT-Vacinas laboratory according to the following guidelines. RT-qPCR tests for SARS-CoV-2 were performed with RNA purified from nasopharyngeal swab samples. RNA was extracted from 140 µL of samples using the QIAamp Viral RNA Mini Kit (Qiagen, Hilden, Germany,), according to protocols provided by the manufacturer. RT-qPCR was performed using primers and probes described in the Berlin (Charité/Berlin, Germany) protocol [8], targeting the gene E from SARS-CoV-2 and the human RNAse P mRNA, used as endogenous amplification reaction control. Reactions were carried out with the Promega GoTaq^®^ Probe 1-Step RT-qPCR Kit (Promega, Charbonnières-les-Bains, France) according to manufacturer’s recommendations, and the QuantStudio™ 5 Real-Time PCR System (Thermo Fisher, Waltham, MA, USA). To determine the lower limit of detection, the primer–probe sets were tested using purified SARS-CoV-2 RNA diluted in RNA elution buffer. The samples were assigned as undetectable when no SARS-CoV-2 gene E amplification occurs or a $C_{t}$ value above 37 in individual tests was obtained. Pool samples were prepared by mixing individual samples to a final volume of 140 μL before RNA extraction.

### 2.6. Calibration Experiments

The calibration phase of this study was performed using samples that were collected from patients with severe symptoms, which were processed individually. To determine a lower bound for γ in the specific setting used in this study, 14 samples with known $C_{t}$ values were diluted in pools of sizes 16 and 32, and Formula (3) was employed in each case. The resulting values of γ were adjusted by a lognormal distribution. The value $\bar{\gamma }=\;1.46$ corresponds to the 90% percentile of that distribution, meaning that $Pr\left(\gamma \geq \bar{\gamma }\right)=0.9$.

The variability of the apparent values between different executions of the testing procedure in the same experimental apparatus depends on several factors, such as the accuracy of the volume measurements made during the preparation of the pools. Although this study did not examine variations considering different experimental settings, it is reasonable to expect that the use of different reagents, for example, will also cause differences. For this reason, it is recommended that each laboratory that uses the procedures proposed in this article carry out a calibration test similar to the one described here. Notice that the calibration assay is quite simple, typically requiring only one batch run of the PCR system with 10 to 20 pooled volumes, which can be performed within a few hours.

### 2.7. Cut-Off Adjustment and Pool Size Choice

In the testing procedures adopted in this study, a range of different target cut-off values for individual samples was examined, with ${\bar{C}}_{t}$ varying from ${\bar{C}}_{tmin}=33$ to ${\bar{C}}_{tmax}=37$ respectively, leading to the following range of instances of Equation (4) for cut-off values to be adopted as functions of the pool size N:

(6) $$\begin{array}{c} {\bar{C}}_{tmin}^{*}=33+2.643\;\ln\left(N\right)\\ {\bar{C}}_{tmax}^{*}=37+2.643\;\ln\left(N\right) \end{array}$$

Those different target values mean that ${\bar{C}}_{tmax}^{*}$ should be used whenever the detection of samples with individual $C_{t}$ up to 37 is considered relevant, while ${\bar{C}}_{tmin}^{*}$ may be used if not detecting samples with individual $C_{t}$ above 33 is assumed to be admissible.

In the case of the laboratory setting employed in this study, the upper limit of the detection range is $C_{M}$ = 41. The following upper bounds for the pool size, ${\bar{N}}_{33}$ and ${\bar{N}}_{37}$, respectively corresponding to ${\bar{C}}_{tmin}=33$ and ${\bar{C}}_{tmax}=37$, are calculated from Equation (5):

(7) $$\begin{array}{c} {\bar{N}}_{33}\;=\;20.65\\ {\bar{N}}_{37}\;=\;4.54 \end{array}$$

**Figure 1:**
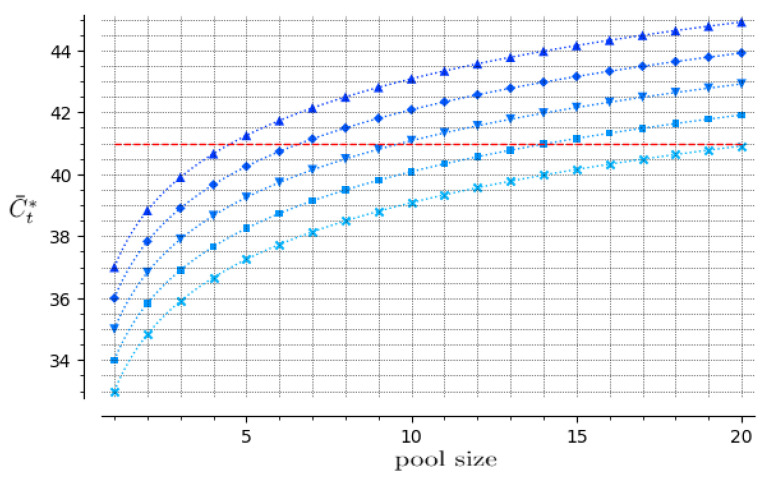
Cut-off values, ${\bar{C}}_{t}^{*}$, that are to be employed in pool testing, as a function of pool size *N*, for individual test cut-off values ${\bar{C}}_{t}=33$ (x), ${\bar{C}}_{t}=34$ (■), ${\bar{C}}_{t}=35$ (▼), ${\bar{C}}_{t}=36$ (♦), and ${\bar{C}}_{t}=37$ (▲). The maximal possible value for the adjusted cut-off in pool processing, ${\bar{C}}_{t}^{*}=41$, is represented by the horizontal dashed line. The maximum pool sizes are N = 4, for ${\bar{C}}_{t}=37$, and N = 20, for ${\bar{C}}_{t}=33$, both allowing ${\bar{C}}_{t}^{*}\;<\;41$.

Equations (6) and (7) represent the guiding information for the adaptation of testing procedures for pool testing. After choosing the target cut-off value for individual testing within the range ${\bar{C}}_{tmin}=33$ to ${\bar{C}}_{tmax}=37$, Equation (6) shows the correction to be performed in the pool cut-off value ${\bar{C}}_{t}^{*}$ for discriminating between positive pools and negative ones. Equation (7) shows the respective upper bounds for the pool sizes that still allow the detection of pools containing one positive sample on the edge of detectability. Those relations are represented in Figure 1.

### 2.8. Pool Testing for Patients with Mild Symptoms

From 9 September to 10 October 2020, adults attending a public emergency care unit, reference for COVID-19 in the city of Belo Horizonte, Brazil, and presenting flu-like symptoms were invited to participate. Those who agreed to participate (*n* = 220) signed an informed consent form, answered a questionnaire reporting clinical and socioeconomic aspects, and had nasopharyngeal swab material collected. The collected material was sent to CT-Vacinas for the execution of testing procedures.

The 220 samples were grouped into 40 pools, 23 of them were positive and 11 pools presented a single positive sample, corresponding to 50 patients. Those patients’ age ranges from 20 to 88 years (40 ± 16 years) and most of them were female (59%). Their symptoms had started from 1 to 15 days before the test (6 ± 4 days) and half of them had no comorbidities. Individual $C_{t}$ values ranged from 12.2 to 37.1, and 50% of the patients showed $C_{t}$ values up to 20.0. The initial pool size of 12 was successively reduced to 8, 4, and 3 to adjust it to the disease prevalence measured during the initial phase of the study. It should be noticed that at the moment when this study started, there was no information about the prevalence of the disease among the population of Belo Horizonte. The initial pool size of 12 was chosen assuming a prevalence figure under 1%. However, the results soon revealed a prevalence between 15% and 25% of COVID-19 in the patients with flu-like symptoms that attended that care unit. This led to the resizing of pools to keep the optimum size from an economic point of view. It is important to stress that pooling was random, and positive samples were not purposely included in pools.

## 3. Results

Among 23 positive pools, 11 pools presented a single positive sample. The pools with more than one detectable sample were not included in this study, as they do not follow Equations (2) and (3), since those equations assume the worst case in which one positive sample is diluted such that the viral RNA concentration is decreased by a factor of 1/*N*. Table 1 shows $C_{t}$ values of those individual samples as well as $C_{t}$ values obtained with each pool and the recommended increase in cut-off value (see also Appendix A). A recommended increase in cut-off values relative to the RT-qPCR original cut-off is calculated in order to compensate dilution caused by pooling. This increase in $C_{t}$ ranges from 6.6 in polls of 12 samples containing one positive sample, to 2.9 in polls with 3 samples containing one positive sample.

## 4. Discussion

All empirical increases in $C_{t}$ values, presented in Table 1, were smaller than the recommended increase in the cut-off. This outcome was expected since the adapted cut-off value was calculated from a lower bound estimate of the amplification factor γ. Not-withstanding, the excess of the recommended increase in relation to the empirical increase was relatively small in most cases. It should be noticed that, although it is expected that different experimental settings will not lead to recommended increases in cut-off values that are too different from the ones presented in Table 1, a prudent approach would be to carry out calibration experiments in each laboratory that would run pooled testing programs, at least until more data is available on the variation of the apparent amplification factor γ in different experimental contexts.

The proposed relaxation in cut-off is dependent on the pool size, allowing a relatively tight correction to avoid loss of detection of positive samples such as the ones presented in the 4th and 6th lines of Table 1. If the usual fixed cut-off ${\bar{C}}_{t}$ = 37 was employed in the pool processing, the pools containing those samples would have been considered negative. The proposed method also avoids excessively large corrections that could result in several false-positive pools.

The choice of a target cut-off ${\bar{C}}_{t}$ should be done based on an assessment of the risk of not detecting infective individuals as smaller values are adopted. A review article [9] asserts that an increasing body of evidence suggests that $C_{t}$ values are useful proxies for infectivity and discusses some studies that attempted to cultivate the virus from samples with different $C_{t}$ values. Those studies have found that viral culture was largely unsuccessful when $C_{t}\;>\;33$. Reference [10] reports an experiment with more than 3000 samples in which less than 3% of the ones with $C_{t}>35$ presented virus that could be cultivated. Higher target values are more conservative: the value ${\bar{C}}_{t}=37$ has been used by CT-Vacinas laboratory in most of the individual tests that have been performed in the last months because it represents a good compromise between the objectives of detecting all infected individuals and avoiding false-positive results. In the case of pool testing, the choice of the target cut-off ${\bar{C}}_{t}$ causes a major impact in the cost-effectiveness outcomes, since different choices in the range 33–37 lead to maximum pool sizes that vary from 4 to 20, as shown in Equation (7) and Figure 1. A specific ${\bar{C}}_{t}$ value should be chosen according to the purpose of the testing procedure, which will determine the acceptable risk level.

As the cut-off value ${\bar{C}}_{t}^{*}$ to be adopted in a pool testing is increased in relation to the individual testing cut-off ${\bar{C}}_{t}$, it should be expected that the rate of false-positive results in pool testing also increases. In fact, among the pools that were processed in this study, there was one pool of size N = 3 which presented a positive result with $C_{t}^{*}=\;38.7$, although the individual testing of all samples resulted negative. This event illustrates an important property of the proposed methodology: there is no increment in false-positive results in relation to individual testing, due to the final phase of individual testing of all samples whenever a pool is found to be positive.

It is worthy to comment that the optimal pool sizes, under the viewpoint of cost optimization, will depend on the prevalence of the disease in the population under study. As shown in Reference [11], the optimal pool sizes may vary from 12, for prevalence under 1%, to 3, for prevalence between 13% and 30%. For prevalence of 1%, the expected savings are nearly 80%, while in the case of prevalence of 13%, the expected resource savings are nearly 33%. For a prevalence of 30%, the expected savings are only about 1%, which means that the pool testing technique no longer presents an advantage. In the case of the study reported here, the weekly prevalence varied between 15% and 25%, leading to overall cost savings of approximately 15%.

In summary, the following procedure for the adaptation of $C_{t}$ cut-off value for SARS-CoV-2 detection in pools and for choosing the maximum admissible pool size is proposed here:

- Find an estimate of lower bound for the amplification factor value corresponding to the laboratory setting to be used, by a calibration assay that compares $C_{t}$ of individual and pooled samples, using Equation (3) and a lognormal parametric model of probability distribution.
- For each pool size, calculate the corresponding correction of cut-off value, according to Equation (4).
- Considering the equipment detection range and the desired target cut-off for individual testing, employ Equation (5) for finding the maximal admissible pool size.
- If the optimal pool size under the economic viewpoint, as stated in Reference [11], is greater than that maximal admissible pool size, adopt that maximal value; otherwise, employ the optimal size.

The proposed procedure enhances the consistency of RT-qPCR pool testing by enforcing that the scales of detectability in pool processing and in individual sample processing are compatible. This procedure may contribute to reduce false-negative results in RT-qPCR pool testing, enhancing its contribution to large-scale testing for COVID-19.

## Figures and Tables

**Table 1:** $C_{t}$ values in pools with a single positive sample and recommended increase in cut-off value.

Pool Size	$C_{t}$ Value of Individual Sample ^1^	$C_{t}$ Values Obtained with Each Pool	Empirical Increase of $C_{t}$	Recommended Increase in Cut-Off Value: $\left(\frac{\ln\left(N\right)}{\ln\left(\bar{\gamma }\right)}\right)$
*N*	$C_{t}$	$C_{t}^{*}$	${∆}_{e}$	
12	16.9	20.7	3.8	6.6
8	18.1	23.1	5.0	5.5
8	21.0	24.3	3.3	5.5
8	35.4	40.5	5.1	5.5
8	26.4	28.1	1.7	5.5
3	34.8	37.5	2.7	2.9
3	27.5	28.9	1.4	2.9
3	16.6	18.2	1.6	2.9
3	30.8	33.5	2.7	2.9
3	22.6	25.0	2.4	2.9
3	22.5	24.9	2.4	2.9

Note: ^1^ a single sample was positive in each analyzed pool.

## Data Availability

All data will be publicly available.

## Appendix A

**Table A1:** Characterization of pools and individual samples tested in the study.

#	Sample ID	Patient Initials	Age	Gender	Sample Collection Date	Sample Receipt Date	Pool #	Pool Size	Pool Plate ID	Pool Reaction Date	Pool Gene E Ct Value	Pool RNase P Ct Value	Pool RT-qPCR Result	Individual Plate ID	Individual Reaction Date	Individual Gene E Ct Value	Individual RNase P Ct Value	Individual Result
1	**P-A1**	AOD	38	Female	10 September 2020	11 September 2020	1	12	P332	11 September 2020	20.7	23.4	Detected	P336	14 September 2020	37.4	22.0	Undetected
2	**P-A2**	FOH	25	Male	10 September 2020	11 September 2020								P336	14 September 2020	Undetermined	22.9	Undetected
3	**P-A3**	FDM	67	Female	10 September 2020	11 September 2020								P336	14 September 2020	Undetermined	22.4	Undetected
4	**P-A4**	SMR	27	Male	10 September 2020	11 September 2020								P336	14 September 2020	Undetermined	22.9	Undetected
5	**P-A5**	MSS	47	Female	10 September 2020	11 September 2020								P336	14 September 2020	16.9	22.3	Detected
6	**P-A6**	FCN	38	Male	10 September 2020	11 September 2020								P336	14 September 2020	Undetermined	23.0	Undetected
7	**P-A7**	MMG	37	Male	10 September 2020	11 September 2020								P336	14 September 2020	Undetermined	21.2	Undetected
8	**P-A8**	ISN	30	Female	10 September 2020	11 September 2020								P336	14 September 2020	Undetermined	21.2	Undetected
9	**P-A10**	WASM	32	Male	10 September 2020	11 September 2020								P336	14 September 2020	Undetermined	26.8	Undetected
10	**P-A11**	ACRA	56	Female	10 September 2020	11 September 2020								P336	14 September 2020	Undetermined	22.4	Undetected
11	**P-A12**	FFD	62	Female	10 September 2020	11 September 2020								P336	14 September 2020	Undetermined	24.6	Undetected
12	**P-A13**	JSF	32	Male	10 September 2020	11 September 2020								P336	14 September 2020	Undetermined	24.3	Undetected
77	**P-A105**	VFC	48	Female	22 September 2020	23 September 2020	8	8	P354	23 September 2020	23.1	25.5	Detected	P362	28 September 2020	Undetermined	26.6	Undetected
78	**P-A106**	PMAA	42	Female	22 September 2020	23 September 2020								P355	24 September 2020	Undetermined	25.8	Undetected
79	**P-A107**	FSC	31	Female	22 September 2020	23 September 2020								P355	24 September 2020	Undetermined	26.7	Undetected
80	**P-A108**	EPAF	34	Male	22 September 2020	23 September 2020								P355	24 September 2020	Undetermined	25.8	Undetected
81	**P-A109**	LPSJ	22	Male	22 September 2020	23 September 2020								P355	24 September 2020	Undetermined	23.1	Undetected
82	**P-A110**	CORA	19	Female	22 September 2020	23 September 2020								P355	24 September 2020	Undetermined	26.5	Undetected
83	**P-A111**	RSP	20	Female	22 September 2020	23 September 2020								P355	24 September 2020	Undetermined	26.5	Undetected
84	**P-A112**	AGPJM	39	Male	22 September 2020	23 September 2020								P355	24 September 2020	18.1	25.5	Detected
109	**P-A146**	SCJ	34	Male	25 September 2020	28 September 2020	14	3	P362	28 September 2020	28.9	25.2	Detected	P365	29 September 2020	Undetermined	25.0	Undetected
110	**P-A147**	AOMC	34	Male	25 September 2020	28 September 2020								P365	29 September 2020	Undetermined	25.5	Undetected
111	**P-A148**	AROA	25	Female	25 September 2020	28 September 2020								P365	29 September 2020	27.5	25.3	Detected
112	**P-A149**	DQM	36	Male	25 September 2020	28 September 2020	15	3	P362	28 September 2020	18.2	24.6	Detected	P365	29 September 2020	Undetermined	24.5	Undetected
113	**P-A150**	SPK	25	Female	25 September 2020	28 September 2020								P365	29 September 2020	16.6	25.0	Detected
114	**P-A151**	FMM	65	Female	25 September 2020	28 September 2020								P365	29 September 2020	Undetermined	24.9	Undetected
118	**P-A191**	DCD	61	Male	30 September 2020	1 October 2020	29	3	P369	1 October 2020	25.0	25.3	Detected	P372	2 October 2020	Undetermined	28.1	Undetected
119	**P-A192**	RBR	61	Female	30 September 2020	1 October 2020								P372	2 October 2020	Undetermined	25.8	Undetected
120	**P-A193**	NAA	38	Female	30 September 2020	1 October 2020								P372	2 October 2020	22.6	25.3	Detected
127	**P-A200**	CSC	45	Female	2 October 2020	5 October 2020	32	3	P374	5 October 2020	33.5	28.4	Detected	P375	6 October 2020	Undetermined	28.5	Undetected
128	**P-A201**	JF	41	Male	2 October 2020	5 October 2020								P375	6 October 2020	30.8	29.3	Detected
129	**P-A202**	FFI	73	Male	2 October 2020	5 October 2020								P375	6 October 2020	Undetermined	28.0	Undetected
130	**P-A203**	SOE	24	Female	2 October 2020	5 October 2020	33	3	P374	5 October 2020	24.9	29.2	Detected	P375	6 October 2020	Undetermined	28.9	Undetected
131	**P-A204**	FSA	44	Female	2 October 2020	5 October 2020								P375	6 October 2020	Undetermined	28.4	Undetected
132	**P-A205**	MSN	44	Male	2 October 2020	5 October 2020								P375	6 October 2020	22.5	28.4	Detected
133	**P-A209**	KTSP	32	Female	2 October 2020	5 October 2020	35	8	P375	6 October 2020	24.3	28.3	Detected	P378	7 October 2020	Undetermined	30.3	Undetected
134	**P-A210**	SR	53	Male	2 October 2020	5 October 2020								P378	7 October 2020	Undetermined	27.9	Undetected
135	**P-A211**	FCGBVC	26	Female	5 October 2020	6 October 2020								P378	7 October 2020	Undetermined	28.0	Undetected
136	**P-A212**	PASA	36	Female	5 October 2020	6 October 2020								P378	7 October 2020	21.0	29.1	Detected
137	**P-A213**	DSJ	35	Male	5 October 2020	6 October 2020								P378	7 October 2020	Undetermined	30.0	Undetected
138	**P-A214**	FGVG	26	Male	5 October 2020	6 October 2020								P378	7 October 2020	Undetermined	30.8	Undetected
139	**P-A215**	GSMM	29	Male	5 October 2020	6 October 2020								P378	7 October 2020	Undetermined	28.5	Undetected
140	**P-A216**	RSM	45	Female	5 October 2020	6 October 2020								P378	7 October 2020	Undetermined	28.3	Undetected
141	**P-A217**	GCJ	37	Male	5 October 2020	6 October 2020	36	8	P375	6 October 2020	40.5	27.5	Detected	P378	7 October 2020	Undetermined	30.2	Undetected
142	**P-A218**	AFS	35	Female	5 October 2020	6 October 2020								P378	7 October 2020	Undetermined	29.6	Undetected
143	**P-A219**	LAL	63	Female	5 October 2020	6 October 2020								P378	7 October 2020	Undetermined	27.8	Undetected
144	**P-A220**	PESM	27	Male	5 October 2020	6 October 2020								P378	7 October 2020	Undetermined	27.5	Undetected
145	**P-A221**	CBSL	29	Female	5 October 2020	6 October 2020								P378	7 October 2020	Undetermined	26.7	Undetected
146	**P-A222**	VASM	20	Male	5 October 2020	6 October 2020								P378	7 October 2020	Undetermined	27.0	Undetected
147	**P-A223**	ARM	31	Male	5 October 2020	6 October 2020								P378	7 October 2020	35.4	27.2	Detected
148	**P-A224**	SMP	29	Female	5 October 2020	6 October 2020								P378	7 October 2020	Undetermined	25.6	Undetected
149	**P-A225**	SPS	22	Female	5 October 2020	6 October 2020	37	8	P378	7 October 2020	28.1	27.7	Detected	P379	8 October 2020	38.7	28.6	Undetected
150	**P-A226**	ASC	46	Female	6 October 2020	7 October 2020								P379	8 October 2020	Undetermined	27.7	Undetected
151	**P-A227**	SMW	20	Male	6 October 2020	7 October 2020								P379	8 October 2020	Undetermined	28.7	Undetected
152	**P-A228**	SCW	28	Male	6 October 2020	7 October 2020								P379	8 October 2020	Undetermined	28.5	Undetected
153	**P-A229**	FSK	31	Female	6 October 2020	7 October 2020								P379	8 October 2020	26.4	28.9	Detected
154	**P-A230**	ROSW	46	Male	6 October 2020	7 October 2020								P379	8 October 2020	Undetermined	28.9	Undetected
155	**P-A231**	PSG	22	Male	6 October 2020	7 October 2020								P379	8 October 2020	Undetermined	29.5	Undetected
156	**P-A232**	JSCM	45	Female	6 October 2020	7 October 2020								P379	8 October 2020	Undetermined	27.0	Undetected
161	**P-A261**	OCH	37	Male	9 October 2020	13 October 2020	43	4	P384	13 October 2020	22.1	28.3	Detected	P385	14 October 2020	18.0	27.9	Detected
162	**P-A262**	MCF	36	Female	9 October 2020	13 October 2020								P385	14 October 2020	Undetermined	26.5	Undetected
163	**P-A263**	GRE	41	Male	9 October 2020	13 October 2020								P385	14 October 2020	Undetermined	28.9	Undetected
164	**P-A264**	NTC	26	Female	9 October 2020	13 October 2020								P385	14 October 2020	Undetermined	29.7	Undetected
165	**P-A265**	ENL	34	Male	9 October 2020	13 October 2020	44	4	P384	13 October 2020	22.0	27.9	Detected	P385	14 October 2020	18.5	25.4	Detected
166	**P-A266**	CPD	42	Female	9 October 2020	13 October 2020								P385	14 October 2020	Undetermined	27.0	Undetected
167	**P-A267**	MOG	70	Male	9 October 2020	13 October 2020								P385	14 October 2020	Undetermined	30.0	Undetected
168	**P-A268**	ABON	31	Male	9 October 2020	13 October 2020								P385	14 October 2020	Undetermined	29.9	Undetected
169	**P-A674**	RLA	42	Female	30 November 2020	1 December 2020	177	3	P468	1 December 2020	37.5	26.6	Detected	P470	2 December 2020	Undetermined	24.6	Undetected
170	**P-A675**	PBM	34	Male	30 November 2020	1 December 2020								P375	2 December 2020	Undetermined	27.6	Undetected
171	**P-A676**	PHS	35	Male	30 November 2020	1 December 2020								P375	2 December 2020	34.8	26.9	Detected
